# Full utilization of noble metals by atom abstraction

**DOI:** 10.1093/nsr/nwaf500

**Published:** 2025-11-13

**Authors:** Kuo Liu, Tao Zhang

**Affiliations:** Institute of Process Engineering, Chinese Academy of Sciences, China; Dalian Institute of Chemical Physics, Chinese Academy of Sciences, China

Fully exposed noble metals enable maximal atom efficiency and play a pivotal role in industrial catalysis, which is indispensable for advancing a sustainable future. To date, different methods, e.g. utilizing single-atom catalysis and controlling nanoparticle (NP) positioning, have been developed to maximize the utilization of noble metals [1–3]. However, active sites created based on the methods above are confronted with heterogeneity, lower stability etc., restricting their application in industrial processes [4].

**Figure 1.**
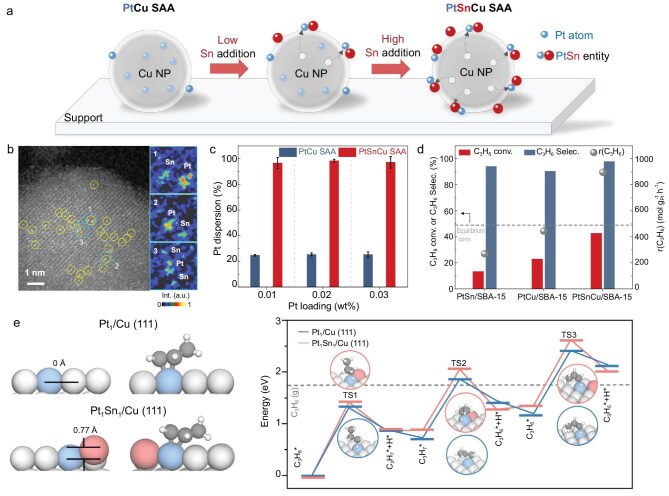
(a) Schematic illustration depicting the structural changes induced by the addition of Sn to PtCu single-atom alloy (SAA). (b) Aberration-corrected high-angle annular dark field scanning transmission electron microscopy (AC-HAADF-STEM) image of Pt_0.1_Sn_0.6_Cu_10_/SBA-15 with pseudo-color images depicting representative areas. (c) Comparison of the Pt dispersion on Cu NPs between the PtCu and PtSnCu catalysts with the Pt loadings of 0.01–0.03 wt%. (d) Comparison of initial PDH performance of PtSn, PtCu and PtSnCu supported on SBA-15. (e) Density functional theory (DFT) calculation results of PDH on the PtCu and PtSnCu catalysts. Reproduced from Sun *et al.* [5] with permission.

To solve the problems above, Professor Jinlong Gong’s group at Tianjin University realized the construction of fully exposed Pt_1_ single atoms through an atom abstraction approach, which ingeniously harnesses the strong interaction between larger and smaller atoms in the host to promote the migration of single atoms from the bulk to the surface [5]. Pt and Cu were co-impregnated over silica or SBA-15 and reduced by H_2_, producing Cu NPs with Pt mainly dispersed in the bulk at low loadings of Pt. However, adding Sn to the PtCu alloy drove Sn to the surface due to the larger atomic radius of Sn relative to Cu, and the strong interaction between Sn and Pt abstracted Pt from the bulk, resulting in the production of Pt_1_Sn_1_ dimers on the surface of Cu NPs (Fig. 1a and b). Theoretical calculations demonstrate that the stability difference between surface and sub-surface Sn was up to 1 eV, making Sn atoms preferentially located on the surface of Cu. Based on the calculated integrated crystal orbital Hamilton population values, the authors found that the interaction between Sn and Pt was stronger than that of Pt–Cu, Pt–Pt, Sn–Cu, Cu–Cu and Sn–Sn, resulting in Pt segregation toward the Cu surface and maximizing the Pt surface exposure. Pt surface exposure on the PtCu catalysts with the Pt loadings of 0.01–0.03 wt% was 25%–29%, while nearly full Pt exposure (93%–97%) was obtained on the surface of the PtSnCu catalysts (Fig. 1c).

The importance of Pt full exposure has been demonstrated by the excellent performance of the PtSnCu catalysts for propane dehydrogenation (PDH). The PtSnCu catalyst exhibited 2-fold greater propane conversion than the PtCu catalyst and 2–3-fold greater than the commercial (mimic) PtSn catalyst at the same Pt loading of 0.05 wt% (Fig. 1d). Additionally, PtSnCu/SBA-15 with a low Pt loading of 0.02 wt% exhibited comparable propane conversion with the commercial (mimic) PtSn catalyst containing 0.3 wt% Pt. The PtSnCu catalyst maintained stable performance during six reaction–regeneration cycles (total time 144 h) due to the stable configuration of Pt_1_Sn_1_ on Cu NPs. Nevertheless, PtSnCu exhibits higher activation energy of PDH and lower intrinsic dehydrogenation activity than PtCu (Fig. 1e). Pt full exposure compensates for the inferior intrinsic dehydrogenation activity and leads to superior propane conversion on PtSnCu, emphasizing the importance of Pt full exposure.

In summary, Gong and co-workers have developed an effective atom abstraction strategy for synthesis of fully exposed noble metals, and minimal Pt loading could be achieved using atom abstraction for PDH. More importantly, atom abstraction exhibits generality and takes place over different catalysts, e.g. MSnCu (M = Ir or Rh), MInCu (M = Pt, Ir or Rh), PtSnAg and PtInAg catalysts [5]. This innovative strategy facilitates the precise synthesis of fully exposed noble metal catalysts with controllable chemical states of single atoms, promoting sustainable catalysis through reduced noble metal consumption. The atom abstraction concept could further inspire catalyst design for sustainable transformations such as plastic upcycling and biomass conversion, potentially advancing carbon neutrality.
